# Dry Age-Related Macular Degeneration and Dense Deposit Disease: A Histopathological Comparison of Macular and Renal Lesions

**DOI:** 10.7759/cureus.102164

**Published:** 2026-01-23

**Authors:** Malik Ladki, Praveena K Gupta, Lauren Malaya, Marjan Afrouzian

**Affiliations:** 1 School of Medicine, University of Texas Medical Branch at Galveston, Galveston, USA; 2 Department of Ophthalmology and Visual Sciences, University of Texas Medical Branch, Galveston, USA; 3 Department of Otolaryngology, University of Arizona, Tucson, USA; 4 Department of Pathology, University of Texas Medical Branch, Galveston, USA

**Keywords:** age-related macular degeneration, bruch’s membrane, dense deposit disease, drusen, kidney

## Abstract

Background

Dry age-related macular degeneration (AMD) and dense deposit disease (DDD) in the kidney have molecular commonality due to the involvement of the complement system. DDD is a renal disease linked to defects of the complement alternative pathway, and in dry AMD, complement components have been found within drusen. In this study, pathologic features of dry AMD and DDD were studied by light and immunofluorescence microscopy to clarify whether morphologic similarities exist between the two diseases.

Methodology

Sections were obtained from (1) the macula of four patients with dry AMD; (2) renal biopsies of two DDD patients; (3) the macula from healthy eye donors; and (4) a kidney biopsy of a normal human subject. Sections were stained with hematoxylin and eosin, periodic acid-Schiff (PAS), and periodic-acid-methenamine silver for light microscopy and with C3b antisera for fluorescence microscopy.

Results

Changes observed in dry AMD specimens included (1) the presence of numerous drusen containing PAS- and silver-positive material; (2) thickening of the Bruch’s membrane; (3) thickening of prominent PAS-positive intercapillary pillars; and (4) degeneration and dropouts in the choriocapillaris and dilatation and congestion of vessels in the Sattler’s layer. In comparison, changes in the DDD specimens included (1) thickening of the glomerular basement membrane (GBM) and presence of double contours; and (2) mesangial and endocapillary proliferation. C3b was positive in both macular drusen and along the GBM.

Conclusions

Basement membrane changes in dry AMD share morphological similarities with the GBM changes in DDD, and, in both diseases, C3b is deposited, suggesting that the association between the two diseases possibly reflects a common pathogenesis.

## Introduction

Age-related macular degeneration (AMD) is one of the major causes of visual impairment in the elderly, representing a major clinical and public health burden worldwide. The most common form of the disease, dry AMD, is characterized by progressive visual decline associated with the accumulation of extracellular deposits between the Bruch’s membrane (BrM) and the retinal pigmented epithelium (RPE) [1]. These deposits, called drusen, are the hallmark of the disease and contribute to RPE dysfunction and subsequent photoreceptor loss [2,3]. Although numerous risk factors have been linked to the onset and progression of dry AMD, the exact mechanism of drusen’s appearance remains unknown [4-6].

Dense deposit disease (DDD), previously termed membranoproliferative glomerulonephritis type II, is a rare renal disorder caused by dysregulation of the alternative complement pathway [7-9]. This proliferative disease is characterized by the accumulation of deposits within the glomerular basement membrane (GBM) [10], resulting in inflammation, altered filtration, and progressive renal dysfunction [11].

Several patients with DDD also develop deposits along the choriocapillaris-BrM-RPE interface [12,13]. Notably, this interface shares some morphologic and physiologic similarities with the glomerular capillary-GBM-podocyte interface [14]. Over time, these individuals develop visual deficits due to macular atrophy and photoreceptor loss [15]. It is worth noting that histological similarities between the drusen-basement membrane complex from dry AMD patients and the glomerular filtration apparatus, including the GBM, in DDD patients have not been studied previously.

Despite these observations, the histopathologic similarities between drusen-associated changes in dry AMD and basement membrane alterations in DDD have not been systematically examined. Therefore, this study aimed to compare the morphological and pathogenetic features of dry AMD and DDD using light and immunofluorescence microscopy, with a focus on identifying shared patterns of basement membrane alteration and complement-associated pathology.

## Materials and methods

This study was approved by the institutional review board (IRB) at the University of Texas Medical Branch (approval number: 19-0210) and was conducted in accordance with the tenets of the Helsinki Declaration. Due to the nature of the study, patient consent was not required. Given the descriptive nature of the study, masking was not performed during histopathologic assessment.

Human donor eyes

Fresh whole globes of non-AMD (N = 2) and AMD (N = 4) human donors of ages ranging from 74 to 94 years were purchased within 24 hours postmortem from the eye bank at the National Disease Research Interchange and/or Lone Star Lions Eye bank (Table 1). The diagnosis of dry AMD was confirmed by the presence of drusen under the dissecting microscope. After coronal transection through the equator, the anterior segment was removed. The neural retina and the vitreous were also removed carefully with the help of a brush and forceps, leaving behind the RPE cells attached to the sclera. In a few cases, RPE was stripped using a soft brush multiple times with subsequent rinsing in phosphate-buffered saline under the dissecting microscope. A 5.0 mm trephine was then used to punch out tissue from the posterior pole of the macular region for further processing. Posterior segments of the eyes were kept as fresh or fixed in 4% paraformaldehyde at a pH of 7.4 for light microscopy. For immunofluorescence microscopy, the fresh posterior segment of the eyes was embedded in OCT mounting media. The RPE was stripped from several sections to remove the background autofluorescence of lipofuscin and melanolipofuscin [16,17]. For light microscopy, the fixed specimens were then processed following the College of American Pathologists Practical Guide to Specimen Handling in Surgical Pathology in a Tissue-Tek VIP 6 AI tissue processor [18].

**Table 1:** Demographics and clinical and laboratory data of four patients with dry age-related macular degeneration (AMD).

Age (years)	Race	Sex	Number of eyes	Cause of death	Comorbidities
80	Caucasian	Male	1	Pneumonia	Dry AMD, glaucoma, atrial fibrillation, benign prostatic hyperplasia, stroke, expressive aphasia, coronary artery disease, congestive heart failure, chronic obstructive pulmonary disease
86	Caucasian	Female	1	Subdural hematoma	Dry AMD, coronary artery disease, atrial fibrillation, hypertension, hyperlipidemia
94	Caucasian	Female	2	Myocardial infarction	Dry AMD, hypertension, anemia, atrial fibrillation, pacemaker, type II diabetes mellitus, gastrointestinal carcinomas, hysterectomy

Human kidney specimens

Fresh and formalin-fixed paraffin-embedded (FFPE) human kidney needle biopsies from two patients with pathologic diagnosis of DDD and one human subject with normal renal histology were obtained from the Pathology Department of the University of Texas Medical Branch at Galveston, Texas. The FFPE blocks obtained from the eyes and kidneys were cut at 3 µ, and sections were stained with hematoxylin and eosin (H&E), periodic acid-Schiff (PAS), and periodic acid-methenamine silver (PAMS). OCT-embedded fresh tissue from eyes and kidneys was cut at 3 µ and stained with C3 (C3/C3b/C3c polyclonal antirabbit IgG antibody for immunofluorescence by Dako) following the standard procedure for indirect immunofluorescence staining following the College of American Pathologists Practical Guide to Specimen Handling in Surgical Pathology [18]. Specimens were examined under a Nikon (Eclipse C) microscope with light and fluorescence capabilities, including the following specifications on objectives and numerical apertures: 10×/0.30 OFN25 DIC L/N1; 20×/0.50 ∞/0.17 WD2.1; 40×/0.75 OFN25/DIC M/N2. A high-power LED-type light source was used for fluorescence microscopy. The excitation filter spectrum (red curve), exhibiting a high level of transmission (approximately 75%) between 450 and 490 nm with a center wavelength (CWL) of 470 nm, was used. Photographs for both light and immunofluorescence microscopy were captured using the OMAX 18MP USB 3.0 C-Mount Microscope high-resolution color digital camera. White background correction was performed on all light microscopic images using Photoshop. No changes in the color spectrum were made.

## Results

**Figure 1:**
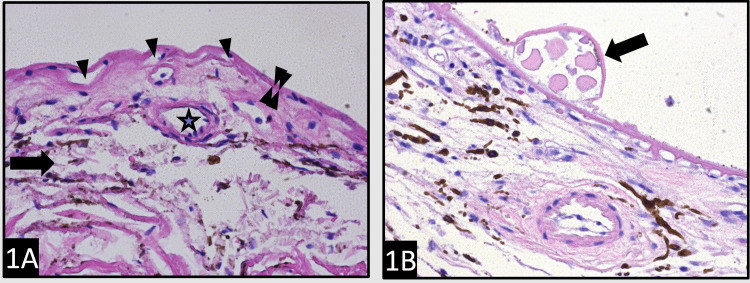
Morphology of the BrM and choriocapillaris: A comparison between normal and dry AMD macula. (A) The normal architecture of the loose choroidal matrix (arrow) is shown. The BrM is thin (double arrowheads) and has a uniform contour. The choriocapillaris (arrowheads) immediately beneath the BrM are evenly spaced. An intermediate vessel of the Sattler’s (star) is also shown (H&E, ×400). (B) AMD RPE-stripped retina showing the presence of a large druse (arrow) containing acellular eosinophilic material. The BrM (double arrowheads) appears thickened (H&E, ×200). AMD = age-related macular degeneration; BrM = Bruch’s membrane; H&E = hematoxylin and eosin; RPE = retinal pigmented epithelium

**Figure 2:**
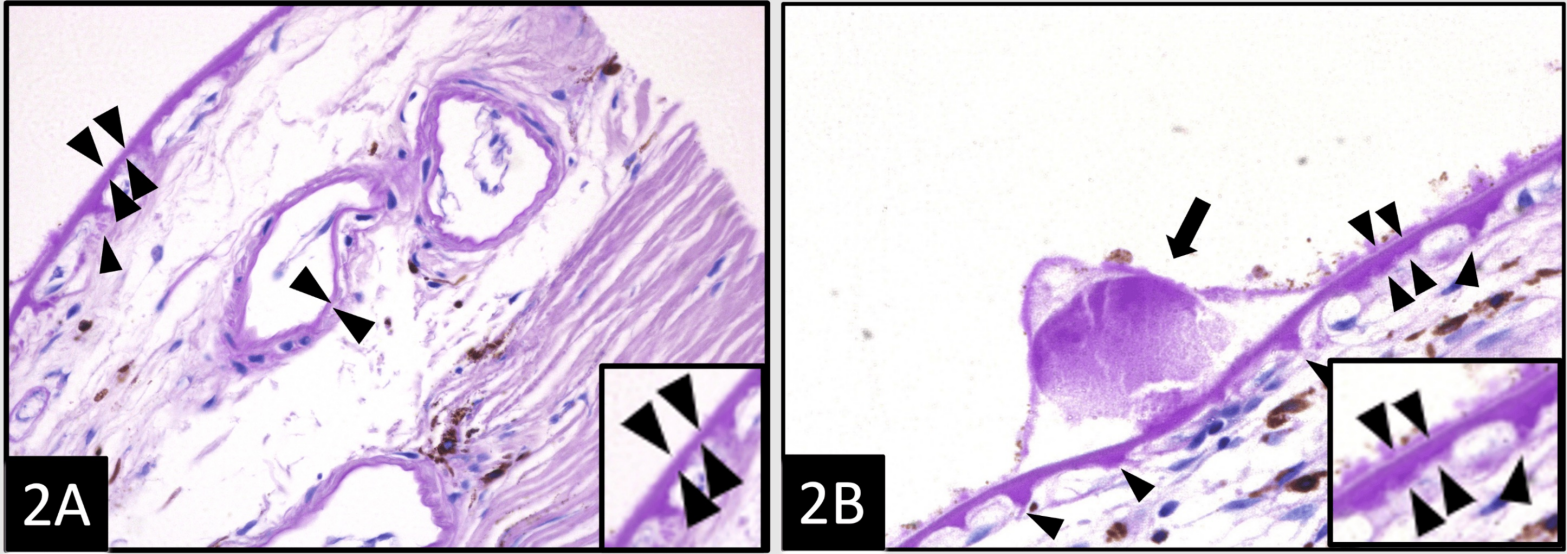
Frequency and size of the intercapillary pillars: A comparison between normal and dry AMD macula. (A) The normal thickness of the BrM (double arrowheads) and the normal thickness of the basal lamina of the intermediate vessel of the Sattler’s layer (arrowheads). One small intercapillary pillar (single arrowhead) is present (PAS, ×400). (B) AMD RPE-stripped macula showing a druse (arrow) containing PAS-positive acellular material. The BrM is thickened (double arrowheads) and multiple PAS-positive intercapillary pillars (single arrowheads) are present (PAS; ×400). AMD = age-related macular degeneration; BrM = Bruch’s membrane; PAS = periodic acid-Schiff; RPE = retinal pigmented epithelium

**Figure 3:**
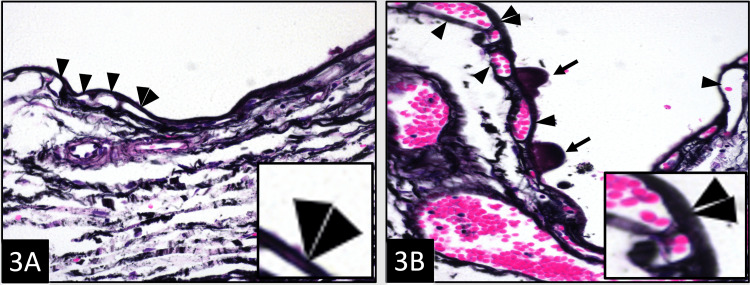
The thickness and contour of the BrM: A comparison between normal eye and dry AMD eye. (A) The normal BrM has a crisp and uniform contour and in areas immediately overlying the choriocapillaris (arrowheads); it appears relatively thin (double arrowhead and inset). No drusen are present. (PAMS, ×400). (B) AMD RPE-stripped macula showing multiple drusen (arrows). The dilated and congested small vessels of choriocapillaris (arrowheads) are observed. The BrM overlying choriocapillaris appears significantly thickened (double arrowheads and inset) (PAMS, ×400). AMD = age-related macular degeneration; BrM = Bruch’s membrane; PAMS = periodic acid-methenamine silver; RPE = retinal pigmented epithelium

Figure 1 shows the normal morphology of the BrM and choriocapillaris after stripping RPE, and compares it to that of the dry AMD macula in an H&E-stained slide. In Figure 1A, the normal architecture of the loose choroidal matrix (arrow) is shown. The BrM is thin (double arrowheads) and has a uniform contour. The choriocapillaris (arrowheads) immediately beneath the BrM are evenly spaced. A medium-sized vessel (star) of the Sattler’s layer is also present. In Figure 1B, the AMD RPE-stripped macula shows the presence of a large druse (arrow) containing acellular eosinophilic material. The BrM (double arrowheads) appears thickened. The frequency and size of the intercapillary pillars are shown in Figure 2, in which a comparison is made between the normal and dry AMD macula when stained with PAS. The normal thickness of the BrM (double arrowheads) and the normal thickness of the basal lamina of the intermediate vessel (arrowheads) of Sattler’s layer are shown in Figure 2A. On the other hand, Figure 2B illustrates the AMD RPE-stripped macula showing a druse (arrow) containing PAS-positive acellular material. The BrM is thickened (double arrowheads), and multiple PAS-positive intercapillary pillars of choriocapillaries (single arrowheads) are noted due to the degeneration of choriocapillaries adjacent to the drusen. The thickness and contour of the BrM stained with PAMS are illustrated in Figure 3 to compare the normal eye with the dry AMD eye. The normal BrM shown in Figure 3A has a crisp and uniform contour, as shown in areas immediately overlying the choriocapillaris (arrowheads), and appears relatively thin (double arrowhead and inset). No drusen is present. In contrast, in Figure 3B, the AMD RPE-stripped macula with multiple drusen (arrows) is shown. The dilated and congested small vessels of choriocapillaris (arrowheads) are also present. The BrM overlying choriocapillaris appears significantly thickened (double arrowheads and inset). A comparative summary of the morphological and pathogenetic features observed in dry AMD versus DDD is presented in Table 2.

**Table 2:** A comparative summary of the morphological and pathogenetic features observed in dry AMD versus DDD. AMD = age-related macular degeneration; DDD = dense deposit disease; GBM = glomerular basement membrane; RPE = retinal pigmented epithelium

Feature category	Dry AMD	DDD	Comparative notes
Deposit characteristics	Drusen with variable size/composition	Dense deposit within the Bruch’s membrane	Both show complement-associated deposits but differ in location/structure
Complement involvement	C3, C5b-9 deposition is common	Marked C3/C5b-9 deposition within GBM and ocular tissues	Similar complement activation pathways are implicated
RPE changes	RPE thinning, pigment migration	RPE loss with basement membrane abnormalities	Both show RPE dysfunction but with distinct morphologies
Bruch’s membrane	Thickening, lipid accumulation	Homogeneous dense deposit replacing Bruch’s membrane layers	Shared basement membrane pathology but with disease-specific patterns
Choroid	Mild thinning	Often normal	Choroidal involvement differs between diseases
Clinical correlation	Gradual central vision decline	Renal involvement + retinal changes	AMD is isolated ocular disease; DDD systemic complement disorder affecting eye

**Figure 4:**
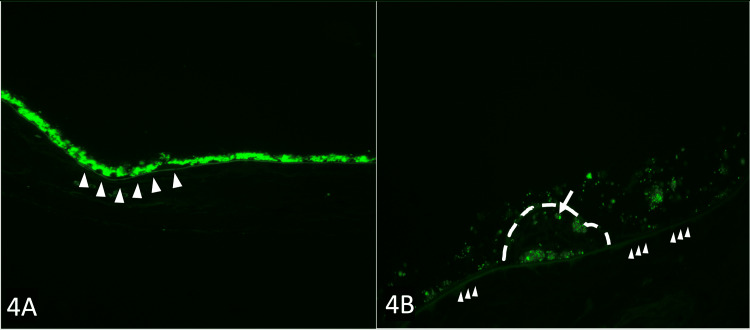
The normal retina and the dry AMD macula stained with C3 by immunofluorescence microscopy. (A) The normal BrM (white arrowheads) shows mild autofluorescence, better shown in the inset (arrow heads). The normal autofluorescence of lipofuscin granules located within the RPE cells is also seen (C3, ×100). (B) Immunofluorescence microscopy of AMD RPE-stripped macula showing the BrM (arrowheads), drusen (dashed line) containing C3-positive material (arrows) (C3, ×400). AMD = age-related macular degeneration; BrM = Bruch’s membrane; RPE = retinal pigmented epithelium

C3 complement staining by immunofluorescence is shown in the normal macula and is compared to the C3 staining in the dry AMD retina (Figure 4). In Figure 4A, the normal BrM (white arrowheads) shows mild autofluorescence, better shown in the inset (arrowheads). This mild autofluorescence of the BrM is considered negative C3 staining. The RPE cells also show bright autofluorescence, which is a normal finding in RPE cells and is due to lipofuscin granules. Therefore, a normal macula is considered negative for C3. The BrM (arrowheads), drusen (dashed line) containing C3-positive material (arrows) are present.

**Figure 5:**
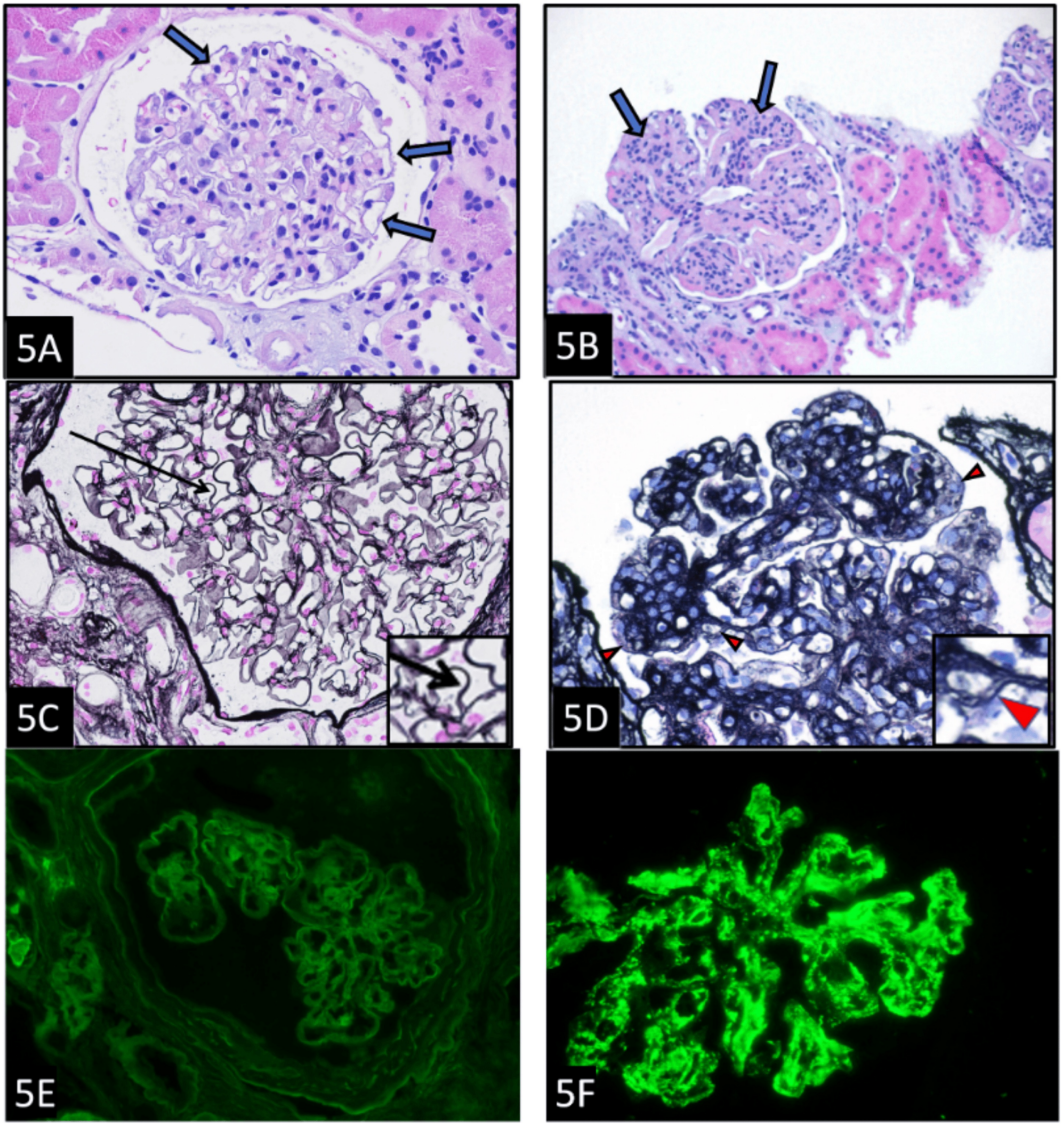
Glomerular morphology: A comparison between normal and DDD kidney. (A) A normal renal glomerulus with patent peripheral capillaries (arrows) and no increase in cellularity (H&E, ×400). (B) DDD glomerulus with obliteration of peripheral capillary loops (arrows) by a cellular proliferation and thickening of the capillary wall (H&E, ×200). (C) The GBM has a crisp, smooth, and uniform contour (arrow) and is composed of one layer (PAMS, ×400). (D) DDD glomerulus showing presence of double contours at the periphery of the lobules (arrowheads) (PAMS, ×400). (E) The normal glomerulus shows only mild autofluorescence (considered negative) in a pseudolinear pattern along the GBM (immunofluorescence microscopy C3, ×400). (F) The DDD glomerulus shows numerous granular and ribbon-like deposits along peripheral capillary loops and within the expanded mesangium (immunofluorescence microscopy C3; ×400). DDD = dense deposit disease; GBM = glomerular basement membrane; H&E = hematoxylin and eosin

The glomerular morphology is presented in Figure 5, and a comparison is made between the normal and DDD kidney. In a normal glomerulus, as shown in Figure 5A, the peripheral capillaries (arrows) are patent, and there is no increase in cellularity. On the other hand, a DDD glomerulus, as shown in Figure 5B, reveals obliteration of peripheral capillary loops (arrows) by cellular proliferation and thickening of the capillary wall. The crisp, smooth, and uniform contour (arrow) of the normal GBM, which is composed of one layer, is shown in Figure 5C. In comparison, a DDD glomerulus shows the presence of double contours at the periphery of the lobules (arrowheads) in Figure 5D. Finally, Figure 5E shows the immunofluorescence morphology of a normal glomerulus that reveals only mild autofluorescence (considered negative) in a pseudolinear pattern along the GBM when stained with C3. A DDD glomerulus, when stained with C3, however, shows numerous granular and ribbon-like deposits along peripheral capillary loops and within the expanded mesangium when stained with C3 (Figure 5F).

## Discussion

Dry AMD and DDD share striking structural, physiologic, and pathogenetic similarities centered on basement membrane pathology [7-9,12,13]. In both conditions, abnormal deposition of extracellular material disrupts specialized filtration interfaces (BrM in the eye and the GBM in the kidney), leading to progressive tissue dysfunction. These shared features indicate common mechanisms underlying disease development despite the distinct clinical manifestations in the retina and kidney.

Micro-anatomic and physiologic similarities

In DDD, electron-dense deposits lead to progressive renal dysfunction, while in dry AMD, drusen within BrM impairs metabolic exchange between the choriocapillaris and the RPE, causing photoreceptor death and vision loss [11,19]. Both membranes share a similar composition of type IV collagen and heparan sulfate arranged as a selectively permeable basement membrane, and act as highly regulated interfaces separating fenestrated capillaries from specialized epithelial cells [11,20]. The similarities between the normal macula of the eye and the normal glomerulus of the kidney, and their associated pathological deposits in dry AMD and DDD, respectively, are represented in Figure 3.

Pathologic similarities

Excess Matrix Production

A prominent shared pathologic feature in both dry AMD and DDD was the presence of excess extracellular matrix. This leads to architectural distortion, which compromises normal charge selectivity and permeability of these specialized filtration interfaces [21,22].

Histochemical Properties

In both dry AMD and DDD, the deposits were PAS-positive, indicating a shared biochemical similarity, which suggests similar pathways of pathogenesis [13].

Electron-Dense Deposits

In dry AMD and DDD, electron-dense material accumulates, respectively, along the BrM and GBM. These deposits share ultrastructural and oligosaccharide features, highlighting a potential common pathobiology underlying extracellular matrix accumulation in both diseases [23].

Pathogenetic similarities

Both drusen in dry AMD and dense deposits in DDD exhibit C3 accumulation along their respective basement membranes, indicating local activation of the alternative complement pathway. This reflects sustained dysregulated activation of the alternative complement pathway, a unifying mechanism that drives tissue injury in both organs [24,25]. Previous studies that have shown C3b positivity in both drusen and DDD have suggested a complement-related pathogenesis. The C3b on unprotected basement membrane surfaces may impair clearance of lipid-rich debris, promoting drusen formation in AMD and dense deposits in DDD [25,26].

Differences between dry AMD and DDD

DDD is a proliferative disease and is associated with cellular proliferation and the influx of inflammatory cells within the glomerulus, while dry AMD lacks these features. This difference reflects organ-specific responses to injury. The glomerular responses to injury are multiple and consist of cellular proliferation, excess matrix formation, sclerosis, and hyalinosis. The BrM’s responses to injury include excess matrix formation along the BrM with increased drusen formation, without cellular proliferation.

Study limitations

This study is based on microscopic observation that specifically aimed at investigating the structural and morphological parallels of dry AMD and DDD. Investigating physiological or functional markers was out of the scope of the study. The small sample size and descriptive design limit generalizability, which precludes any functional or mechanistic conclusion.

## Conclusions

This comparative analysis demonstrates that dry AMD and DDD share a common pattern of basement membrane injury driven by dysregulated alternative complement pathway activation. By drawing direct morphological and pathogenetic parallels between retinal and renal basement membranes, this study advances understanding of drusen formation in dry AMD and provides a strong biologic rationale for the development and application of complement-targeted therapies in this disease.
